# Insights Into the Structural Features, Codon Usage Patterns, and Phylogenetic Analysis in *Neoniphon argenteus* (Teleostei: Holocentriformes) Based on Complete Mitochondrial Genome

**DOI:** 10.1002/ece3.74291

**Published:** 2026-09-04

**Authors:** Lei Xie, Nan Chen, Qi Qiao, Xuedong Xie, Bozhu Huang, Songhui Lu, Yuelei Dong, Lei Cui

**Affiliations:** ^1^ Key Laboratory of Eutrophication and Red Tide Prevention of Guangdong Higher Education Institutes, College of Life Science and Technology, and Southern Marine Science and Engineering Guangdong Laboratory (Zhuhai) Jinan University Guangzhou China; ^2^ Guangdong Ecological and Environmental Monitoring Center Guangzhou China; ^3^ Guangdong Provincial Key Laboratory of Biotechnology for Plant Development, School of Life Sciences South China Normal University Guangzhou China

**Keywords:** codon usage, evolution, Holocentridae, mitogenome, phylogeny, selection pressure

## Abstract

*Neoniphon argenteus* , a widely distributed nocturnal coral reef fish in the family Holocentridae, plays an important role in maintaining coral reef ecosystem health, yet its phylogenetic position remains poorly resolved. To bridge this gap, we sequenced and analyzed the complete mitochondrial genome of a specimen from the South China Sea to characterize its structural features, codon usage patterns, and phylogenetic relationships. The 16,569 bp mitogenome (GenBank: PP190474.1) encodes 13 protein‐coding genes (PCGs), 22 tRNAs, two rRNAs, and two non‐coding regions, exhibiting a distinct A + T bias. All tRNAs fold into typical cloverleaf secondary structures except tRNA‐Ser ^(AGN)^, which lacks the dihydrouridine (DHU) arm. The control region contains palindromic motifs (TACAT/ATGTA) capable of forming hairpin structures and five conserved sequence blocks, whereas the O_L_ region harbors a conserved 5′‐GCCGG‐3′ motif. RSCU analysis revealed 31 frequently used codons (RSCU > 1) with a pronounced preference for A/C‐ending codons. The ΔRSCU method identified 10 candidate optimal codons (GCA, CAA, GAA, GGA, AUU, CUA, CCA, CGA, ACA, and GUC). Selection pressure analysis using EasyCodeML and site‐specific models indicated that all PCGs are predominantly under purifying selection, with no significant evidence of pervasive positive selection. *ND6* exhibited elevated pairwise Ka/Ks ratios (mean = 1.209 ± 0.047), consistent with reduced selective constraint rather than adaptive evolution. Phylogenetic analysis of 19 Holocentriformes species using maximum likelihood and Bayesian inference with partitioned models based on 13 PCGs and two rRNA genes (12S and 16S) assigned all taxa to two well‐supported subfamilies (Holocentrinae and Myripristinae). Within Holocentrinae, *Neoniphon* species form a monophyletic clade nested within a paraphyletic *Sargocentron*, suggesting that the genus *Sargocentron* as currently defined is not monophyletic. This study provides useful baseline molecular data for further exploration of the evolutionary history of *N. argenteus* and other members of Holocentriformes.

## Graphical abstract

This study provides important foundational information for further research on the mitochondrial genomes of Holocentridae.

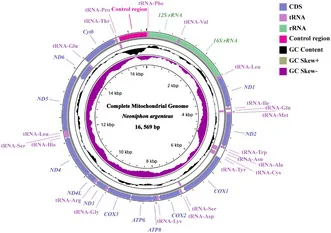

## 1 Introduction

The order Holocentriformes, containing one family Holocentridae (squirrelfish and soldierfish), is a group of primarily nocturnal, benthic and reef‐associated teleost fishes inhabiting tropical and temperate marine habitats worldwide (Nelson et al. 2016). As integral components of coral reef fish assemblages, Holocentriformes significantly influence ecosystem dynamics. Representing a major reservoir of consumer biomass on coral reefs, they mediate energy and nutrient flows (Allgeier et al. 2017). Their larval dispersal across pelagic‐coral reef interfaces (Leis et al. 2011) further facilitates reef replenishment via pelagic productivity (Allgeier et al. 2018). These mechanisms partially resolve the Darwin Paradox, which describes the maintenance of high biodiversity and productivity in nutrient‐poor tropical oceans. While Holocentriformes contribute to coral reef ecosystems, their identification has thus far predominantly relied on morphological characteristics, though this approach faces reliability challenges due to overlapping traits.

In recent years, the mitochondrial genome has gained widespread application in species identification, phylogenetics, and population genetics of teleost fish (Miya et al. 2001; Simon et al. 2006). This is attributed to its maternal inheritance, conserved genome structure, conserved coding content and rare intermolecular genetic recombination (Saccone et al. 1999). Mitogenomic studies have proven particularly valuable for resolving phylogenetic relationships among closely related fish taxa and for understanding patterns of molecular evolution (Tang et al. 2023; Baraf et al. 2024). Recent comparative mitogenomic analyses have provided new insights into the evolutionary dynamics of coral reef fishes, including patterns of codon usage bias, selective pressure, and phylogenetic relationships (Tang et al. 2023; Kundu et al. 2019; Zhang et al. 2024; Tan et al. 2025). These studies have demonstrated that mitogenomic data are particularly useful for resolving phylogenetic relationships at the familial and generic levels in teleosts, and for identifying patterns of molecular adaptation in marine fishes. Recent mitogenomic investigations across diverse fish lineages have further demonstrated the utility of mitochondrial data for resolving phylogenetic relationships and understanding evolutionary dynamics in marine and freshwater fishes (Aini et al. 2025; Kundu et al. 2023, 2024; Wang et al. 2024). To date, out of the 91 known species of Holocentriformes, only 18 complete mitochondrial sequences are available in NCBI, limiting phylogenetic resolution within this ecologically important group.

*N. argenteus* is a widely distributed species within Holocentriformes, ranging in the Indian Ocean‐Pacific Ocean region from Japan to Australia, and is typically found in shallow water (1–19 m) of coral reef areas (Andrews et al. 2023). However, given the significant phenotypic changes observed in reef fishes, including *N. argenteus* , species identification can be challenging. In particular, morphological identification methods often only allow for identification to the genus level, especially during early developmental stages (Leis and Carson‐Ewart 2004). This study presents the first complete mitogenome of *N. argenteus* , determined via Sanger sequencing, along with its gene organization, structural features, codon usage patterns, selective pressure analysis, and phylogenetic relationships. The findings of this research offer new insights for phylogenetic research and enhance our understanding of the mitochondrial genomic characteristics and taxonomy within the order Holocentriformes.

## 2 Materials and Methods

### 2.1 Specimen Acquisition

Mature specimens of *N. argenteus* were collected from the South China Sea (15°47′ N–17°13′ N, 110°49′ E–113°23′ E) in June 2023. The International Union for Conservation of Nature's Red List (version 4.0) did not classify *N. argenteus* as either a protected or endangered species. This study was approved by the Jinan University Application for Laboratory Animal Ethical Review. All experimental procedures complied with international guidelines for laboratory animal care and use. Muscle tissue samples were preserved in 95% ethanol and subsequently stored at −80°C pending analysis. For the specimen voucher, a sample was deposited in the laboratory of the Department of Biology, Jinan University, under deposition number NH2023‐04.

### 2.2 DNA Extraction, Amplification, and Sequencing

Following Proteinase K digestion of dorsal muscle tissue, total genomic DNA was purified employing a specialized animal tissue DNA extraction kit (B518221, SangonBiotect, Shanghai, China). To sequence the mitochondrial genome of *N. argenteus* , 13 primer pairs were developed using conserved regions identified through alignment of the complete mitochondrial genome of *Neoniphon sammara* (GenBank: NC_063501.1) (Table S1). PCR amplification reactions were conducted with Phusion Plus DNA Polymerase (Thermo Fisher Scientific, Waltham, MA, USA) following these conditions: an initial denaturation step at 95°C for 3 min, followed by 35 cycles, each cycle consisting of 20 s at 95°C (denaturation), 45 s at 55°C (annealing), and 1–5 min at 72°C (extension), with a final extension at 72°C for 10 min. Primer walking methodology was applied to conduct bidirectional Sanger sequencing of PCR amplicons on an ABI 3730xl DNA Analyzer (Applied Biosystems, Foster City, CA, USA). Bidirectional Sanger sequencing was performed for all PCR amplicons. The complete mitogenome was assembled without gaps or ambiguous bases, and all overlapping regions between adjacent amplicons showed 100% sequence identity. The original Sanger sequencing chromatograms have been deposited in the Dryad Digital Repository (https://doi.org/10.5061/dryad.vhhmqqp8h).

### 2.3 Complete Mitogenome Analysis

We manually checked and assembled the mitochondrial genome sequence of *N. argenteus* using the Seqman Pro program within the Lasergene software suits (version 17.0, DNAStar Inc., Madison, WI, USA). We annotated the complete sequence using NCBI BLAST (http://blast.ncbi.nlm.nih.gov/Blast, e‐value threshold 1e‐5) and DNA Star software package (version 17.0 DNAStar Inc. Madison, WI, USA). The mitogenome map in this study was produced with the GCView Server using default parameters (Grant and Stothard 2008). The positions of the 13 PCGs and two rRNAs were primarily identified by Dual Organellar Genome Annotator (DOGMA) (Wyman et al. 2004). All tRNA gene sequences were identified using tRNA‐scan‐SE software (version 2.0.12) available at http://lowelab.ucsc.edu/tRNAscan‐SE/, employing the default search mode with “Mito/chloroplast” as the source (Lowe and Eddy 1997). The secondary structures of tRNA genes and O_L_ were delineated using RNA structure software (version 6.4) (Mathews 2014). The analysis of tandem repeat sequences within the control region (CR) was performed using the Tandem Repeats Finder program (version 4.09), which can be accessed at http://tandem.bu.edu/trf/trf.html (Benson 1999). Nucleotide composition skewness was quantified employing the following formulas: A/T‐skew = [(A − T)/(A + T)], G/C‐skew = [(G − C)/(G + C)] (Perna and Kocher 1995). The complete mitogenome DNA sequence of *N. argenteus* has been deposited in the GenBank database under accession number PP190474.1.

### 2.4 Codon Usage Analysis

Codon usage metrics were determined using the web‐based EMBOSS Explorer suite (http://emboss.open‐bio.org/), including: GC content at codon positions 1–3 (GC_1_, GC_2_, GC_3_), total genomic GC (GC_all_), and effective number of codons (ENC). The ENC was used to assess the uneven usage of synonymous codon sets (Wright 1990), with values closer to 20 indicating extreme bias and values closer to 61 indicating low bias. Additionally, CodonW software (version 1.4.4) was used to calculate the relative synonymous codon usage (RSCU). Codons with RSCU > 1 were considered to be used more frequently than expected under equal synonymous codon usage, while those with RSCU < 1 were considered less frequently used (Sharp et al. 1986). Codon usage patterns were analyzed and compared across all 13 PCGs. The vertebrate mitochondrial genetic code (NCBI translation Table 2) was applied throughout the analysis.

To identify putatively optimal codons, we compared RSCU values between two representative genes (*COX2* and *ND6*), following the ΔRSCU method (Sharp et al. 1986). Codons showing a consistent increase in RSCU (ΔRSCU ≥ 0.3) in *COX2* relative to *ND6* were considered candidate optimal codons.

### 2.5 Selection Pressure Analysis

The ratio of nonsynonymous (Ka) to synonymous (Ks) substitution rates (Ka/Ks, also denoted as *ω*) was calculated to evaluate selective pressures acting on the 13 mitochondrial protein‐coding genes (PCGs). Pairwise Ka and Ks values were computed between *N. argenteus* and each of the 18 other Holocentriformes species listed in Table 1 using DnaSP software (version 5.10.01) (Rozas et al. 2003). For each PCG, the mean and standard deviation of Ka/Ks ratios across the 18 pairwise comparisons were calculated.

**TABLE 1:** Summary of the base composition and skewness of whole mitochondrial genomes among 19 Holocentriformes species.

Family	Species (accession number)	Length (bp)	A	T	C	G	A + T	AT skew	GC skew
Holocentrinae	*Sargocentron tiere* (OP035265)	16,573	0.30	0.26	0.29	0.15	0.56	0.08	−0.30
Holocentrinae	*Sargocentron spiniferum* (NC_030597)	16,548	0.30	0.25	0.29	0.15	0.56	0.09	−0.31
Holocentrinae	*Sargocentron melanospilos* (NC_085257)	16,528	0.30	0.25	0.29	0.16	0.55	0.08	−0.31
Holocentrinae	*Sargocentron rubrum* (NC_004395)	16,526	0.30	0.25	0.29	0.16	0.55	0.08	−0.31
Holocentrinae	*Sargocentron bullisi* (OP056898)	16,491	0.30	0.26	0.28	0.16	0.56	0.07	−0.29
Holocentrinae	*Sargocentron diadema* (NC_085256)	16,510	0.30	0.25	0.30	0.16	0.55	0.09	−0.31
Holocentrinae	*Sargocentron microstoma* (OP035193)	16,504	0.30	0.25	0.30	0.15	0.55	0.09	−0.31
Holocentrinae	*Sargocentron punctatissimum* (NC_085258)	16,505	0.30	0.26	0.29	0.15	0.56	0.08	−0.31
Holocentrinae	*Neoniphon opercularis* (NC_085255)	16,639	0.31	0.26	0.28	0.15	0.57	0.09	−0.32
Holocentrinae	*Neoniphon argenteus* (PP190474)	16,569	0.30	0.26	0.28	0.15	0.57	0.08	−0.29
Holocentrinae	*Neoniphon sammara* (NC_063501)	16,741	0.31	0.26	0.28	0.15	0.57	0.08	−0.30
Myripristinae	*Ostichthys japonicus* (NC_004394)	16,541	0.29	0.25	0.30	0.16	0.54	0.08	−0.31
Myripristinae	*Plectrypops lima* (OP035065)	16,560	0.29	0.24	0.30	0.16	0.54	0.09	−0.32
Myripristinae	*Myripristis pralinia* (NC_082848)	16,497	0.29	0.25	0.30	0.16	0.54	0.09	−0.31
Myripristinae	*Myripristis vittata* (NC_063496)	16,520	0.29	0.24	0.30	0.16	0.54	0.09	−0.31
Myripristinae	*Myripristis adusta* (OP035112)	16,535	0.30	0.24	0.30	0.16	0.54	0.10	−0.32
Myripristinae	*Myripristis amaena* (OP035109)	16,534	0.29	0.24	0.31	0.16	0.53	0.09	−0.31
Myripristinae	*Myripristis kuntee* (NC_085252)	16,528	0.29	0.24	0.31	0.16	0.53	0.10	−0.31
Myripristinae	*Myripristis berndti* (NC_003189)	16,531	0.29	0.24	0.31	0.16	0.53	0.10	−0.31

Site‐specific selection analysis was conducted using EasyCodeML (version 1.4) (Gao et al. 2019), a graphical interface for the CodeML program in the PAML package (Yang 2007). Nucleotide sequences of the 13 PCGs from 19 Holocentriformes species were aligned using MUSCLE implemented in MEGA (version 11.0.13) (Tamura et al. 2021) and concatenated into a supermatrix. Site models were applied to allow *ω* to vary among codon sites while assuming a constant *ω* ratio across all branches of the phylogeny. Likelihood ratio tests (LRTs) were performed to compare nested site‐model pairs: M1a (nearly neutral) versus M2a (positive selection), and M7 (beta distribution, *ω* constrained to ≤ 1) versus M8 (beta distribution with an additional site class allowing *ω* > 1). For each LRT, twice the difference in log‐likelihood (2Δℓ) was evaluated against a Chi‐squared distribution with degrees of freedom equal to the difference in the number of parameters (df = 2 for both comparisons). Statistical significance was assessed at *p* < 0.05. When the LRT was significant and the alternative model (M2a or M8) inferred a class of sites with *ω* > 1, the Bayes Empirical Bayes (BEB) method was used to identify specific codons under positive selection, with a posterior probability threshold of ≥ 0.95.

To exclude the possibility that elevated Ka/Ks ratios in *ND6* are an artifact of substitution saturation, we performed saturation tests using DAMBE (Xia et al. 2003). The index of substitution saturation (Iss) for *ND6* was 0.412, which was significantly lower than the critical Iss.c value (0.786) at *p* < 0.001, confirming that substitutions in *ND6* had not reached saturation. Additionally, the use of Sanger sequencing and the absence of Numt‐like sequences in the assembled genome effectively rule out NUMT co‐amplification as a confounding factor.

### 2.6 Phylogenetic Analysis

A total of 19 Holocentriformes mitochondrial genomes were retrieved from GenBank for phylogenetic reconstruction (Table 1). *Rondeletia loricata* (NC_003186) and *Barbourisia rufa* (NC_012046) were used as outgroups, since they represent the most recent sister lineage to Holocentriformes (Johnson et al. 2009). The nucleotide sequences of all 13 protein‐coding genes (PCGs) were aligned separately using Clustal X (version 2.1) with default settings (Thompson et al. 2003) and subsequently concatenated into a supermatrix of 11,430 nucleotides.

To account for heterogeneity in substitution patterns among genes and codon positions, we employed a partitioned analysis strategy. The concatenated dataset was partitioned by gene and by codon position (13 genes × 3 codon positions = 39 partitions). For each partition, the optimal substitution model was selected using ModelTest‐NG (version 0.1.7) (Darriba et al. 2020) based on the Akaike Information Criterion (AIC). The best‐fit models selected for the majority of partitions were GTR + F + I + G4 (for 22 partitions), GTR + F + G4 (for 9 partitions), and TIM2 + F + I + G4 (for 8 partitions). The partitioned analysis yielded a substantially improved model fit (−lnL = 45,673.8) compared to the unpartitioned analysis (−lnL = 48,291.4), with a Δ‐lnL of 2617.6.

As a robustness check, we also performed phylogenetic analyses using (i) the concatenated nucleotide sequences of the 13 PCGs plus the two rRNA genes (12S and 16S)—the 22 tRNA genes were excluded from this analysis because their short length (60–75 bp) and complex secondary structures introduced alignment ambiguity and limited phylogenetic informativeness—and (ii) the deduced amino acid sequences of the 13 PCGs. For the 13 PCGs + 2 rRNAs dataset (15 genes, 13,482 bp in total), we applied a partitioned strategy with 15 partitions (one per gene).

Phylogenetic relationships were inferred using both maximum likelihood (ML) and Bayesian inference (BI) methods. ML analysis was performed with raxmlGUI (version 2.0.10) (Silvestro and Michalak 2012), and node support was evaluated using 1000 bootstrap replicates. Identical gene‐codon partition scheme was applied to Bayesian inference, with substitution model parameters unlinked across each partition for independent estimation. The same partition scheme was applied to Bayesian inference, with substitution model parameters unlinked across partitions to allow independent estimation. BI analysis was conducted with MrBayes (version 3.2.7a) (Ronquist et al. 2012), with four independent Markov Chain Monte Carlo chains run for 10 million generations and sampled every 1000 generations. The first 25% of samples were discarded as burn‐in, and convergence was assessed by ensuring the average standard deviation of split frequencies fell below 0.01.

Phylogenetic analysis was also performed using the deduced amino acid sequences of the 13 PCGs. Amino acid sequences were aligned separately using MAFFT (version 7.453) (Katoh and Standley 2013) with default parameters and concatenated. ProtTest (version 3.4) (Abascal et al. 2005) identified JTT + F + I + G4 as the best‐fit model according to the AIC. ML and BI analyses were conducted using raxmlGUI and MrBayes, respectively, following the same parameters and convergence criteria as described for the nucleotide dataset. All phylogenetic trees were visualized using FigTree (version 1.4.4).

## 3 Results and Discussion

### 3.1 Structural Characteristics

**FIGURE 1:**
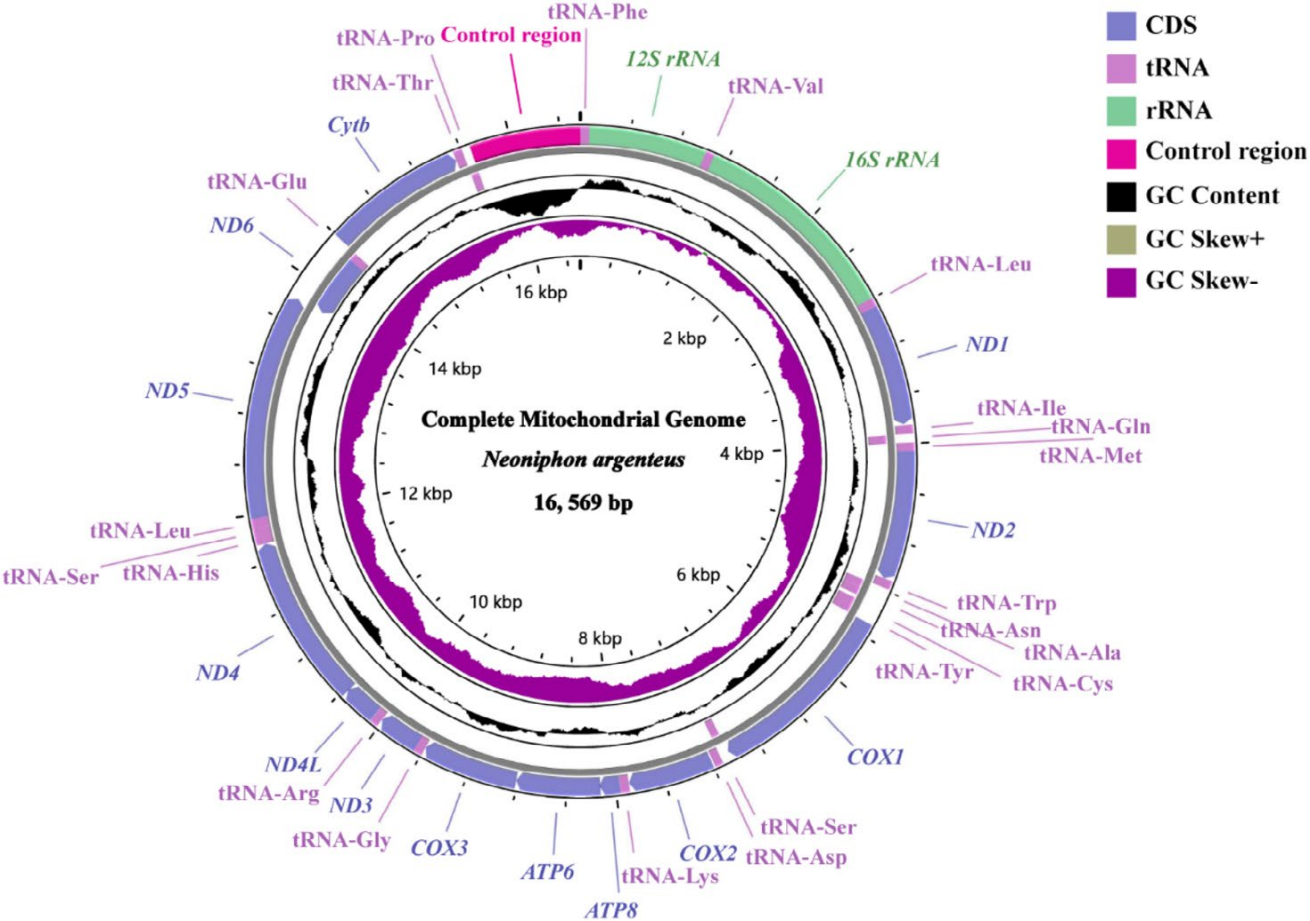
Map of the mitochondrial genomes of *Neoniphon argenteus*. Protein‐coding genes (PCGs) were represented by blue arrows, transfer RNA (tRNA) by lavender arrows, and ribosomal RNA (rRNA) genes by virescent arrows. The tRNA genes were further distinguished by amino acid abbreviations. The black circles represent guanine‐cytosine (GC) content, where outward/inward extensions denote values above/below average. Purple and green circles depict GC skew, with values 0 to 1 shown in purple and −1 to 0 in green. The sequence length was indicated by ticks on the inner cycle.

The mitochondrial genome sequence obtained in this study for *N. argenteus* had a total length of 16,569 bp, falling within the range of lengths (16,491–16,741 bp) reported for 18 other Holocentriformes species (Table 1). The genome composition exhibited a high degree of conservation. All 19 mitochondrial genomes comprised 13 protein‐coding genes (PCGs), 22 tRNAs, 2 rRNAs and two non‐coding regions: control region and origin of the light‐strand (O_L_). Gene annotations were detailed in Figure 1 and Table 2, revealing H‐strand encoding for 28 genes versus light‐strand localization for nine (tRNA‐Asn, tRNA‐Ala, tRNA‐Tyr, tRNA‐Ser ^(UCN)^, tRNA‐Gln, tRNA‐Glu, tRNA‐Cys, tRNA‐Pro and *ND6*). Excluding the control region, there were 11 intergenic spacers (1–31 bp in length), along with seven gene overlaps throughout the *N. argenteus* mitogenome (Table 2). Comparison of Holocentriformes mitogenomes demonstrated exceptional conservation, with minimal intergenic overlaps implying optimization for transcriptional and translational efficiency.

**TABLE 2:** Structure and characteristics of the mitochondrial genome of *Neoniphon argenteus* .

Gene	Coding strand	Position	Spacer (H)/overlap (L)	Start/Stop codon
tRNAL‐Phe	H	1–70		
12S rRNA	H	71–1018	0	
tRNAL‐Val	H	1019–1090	0	
16S rRNA	H	1091–2794	0	
tRNAL‐Leu ^(UAA)^	H	2795–2868	0	
*ND1*	H	2869–3843	0	ATG/TAA
tRNA‐Ile	H	3852–3923	8	
tRNAL‐Gln	L	3923–3993	−1	
tRNA‐Met	H	3993–4063	−1	
*ND2*	H	4064–5110	0	ATG/TAA
tRNA‐Trp	H	5111–5183	0	
tRNA‐Ala	L	5185–5253	1	
tRNA‐Asn	L	5255–5327	1	
tRNA‐Cys	L	5359–5425	31	
tRNA‐Tyr	L	5426–5493	0	
*COX1*	H	5495–7045	1	GTG/TAA
tRNA‐Ser ^(UGA)^	L	7046–7116	0	
tRNA‐Asp	H	7120–7188	3	
*COX2*	H	7201–7892	12	ATG/TA
tRNA‐Lys	H	7893–7966	0	
*ATP8*	H	7968–8135	1	ATG/TAA
*ATP6*	H	8126–8809	−10	ATG/TAA
*COX3*	H	8809–9593	−1	ATG/TAA
tRNA‐Gly	H	9594–9664	0	
*ND3*	H	9665–10,013	0	ATG/T
tRNA‐Arg	H	10,014–10,082	0	
*ND4L*	H	10,085–10,381	2	ATG/TAA
*ND4*	H	10,375–11,755	−7	ATG/T
tRNA‐His	H	11,756–11,824	0	
tRNA‐Ser ^(GCU)^	H	11,825–11,893	0	
tRNA‐Leu ^(UAG)^	H	11,895–11,967	1	
*ND5*	H	11,968–13,806	0	ATG/TAA
*ND6*	L	13,802–14,323	−5	ATG/AGG
tRNA‐Glu	L	14,324–14,392	0	
*Cyt b*	H	14,397–15,537	4	ATG/T
tRNA‐Thr (T)	H	15,538–15,609	0	
tRNA‐Pro (P)	L	15,609–15,678	−1	
Control region	H	15,679–16,569	0	

*Note:* H and L designate heavy and light strand, respectively.

The composition of the mitogenome (A: 30.48%, C: 28.02%, G: 15.37%, T: 26.13%) (Table 1) exhibited a distinct A + T bias. Asymmetry, commonly observed in teleost fishes, demonstrates evolutionary conservation of mitogenomes (Cui et al. 2017). Mitogenomic analysis revealed a positive AT‐skew (0.08) and negative GC‐skew (−0.29) in *N. argenteus* , aligning with other Holocentriformes species (mean AT‐skew: 0.09 ± 0.01; GC‐skew: −0.27 ± 0.11; Table 1). This reveals significant A‐ and C‐base compositional biases in its mitochondrial genome.

### 3.2 Transfer RNA and Ribosomal RNA Genes

In this research, a total of 22 tRNAs were discovered within the mitogenome of *N. argenteus* displaying canonical structural features. These tRNAs with lengths varying from 66 bp in the case of tRNA‐Cys to 74 bp for tRNA‐Leu (Table 2). Among these tRNAs, serine and leucine were transferred by two tRNA respectively (Ser: tRNA‐Ser ^(AGN)^, tRNA‐Ser ^(UCN)^; Leu: tRNA‐Leu ^(UUR)^, tRNA‐Leu ^(CUN)^), while other amino acids were each encoded by a single tRNA gene (Figure S1). All tRNA genes amount to 1554 bp, constituting approximately 9.30% of the complete mitogenome in which 14 tRNA genes were positioned on the H‐strand and the remaining set localized to the L‐strand. All tRNAs fold into characteristic cloverleaf secondary structures. However, an exception was tRNA‐Ser ^(AGN)^, which does not possess a dihydrouridine (DHU) arm (Figure S1). In this particular tRNA, what would be the DHU arm was instead a large loop, rather than the conserved stem and loop structure. This pattern is commonly observed in vertebrate mitochondrial tRNAs and has been documented in numerous fish species (Ohtsuki et al. 2002; Zhang et al. 2024). Research has shown that although tRNA‐Ser ^(AGN)^ possesses a notably simplified structure, it modifies the distance between the 3′‐terminus and the anticodon via a flexible connecting region. This adaptation allows it to perform its function of carrying and transporting amino acids like other tRNAs within the ribosome (Ohtsuki et al. 2002). There were six types of base mismatches among these tRNAs: A‐C, U‐U, A‐A, C‐C, A‐G, C‐U. Mismatching mainly occurred in the aminoacyl acceptor arm and TψC arm, except for the mispairing on the DHU stem (tRNA‐Trp) and anticodon arm (tRNA‐Ser ^(UCN)^) (Figure S1). These mismatches could be corrected through post‐transcriptional RNA editing, which does not cause amino acid transport disorders (Janke and Pääbo 1993).

The mitochondrial genome of *N. argenteus* encompassed the 12S rRNA gene, which was located between the tRNA‐Phe and tRNA‐Val genes. Additionally, the 16S rRNA gene was situated between the tRNA‐Val and tRNA‐Leu genes (Figure 1). These genes exhibited lengths of 948 and 1704 bp, respectively. Both genes were encoded on the H‐strand, with no intergenic regions or overlapping regions. The AT content of rRNAs was 55.21%, and the GC was 44.79%, with the highest proportion of adenine (34.09%) (Table S2), which was consistent with metazoans (Donne‐Goussé et al. 2002).

### 3.3 Protein‐Coding Genes

The combined length of the protein‐coding genes in the *N. argenteus* mitochondrial genome was 11,430 bp, representing 68.98% of the total genome, with a pronounced AT‐bias (A + T = 56.31%) (Table S2), encoding 3800 amino acid residues. Except for the *ND6* gene located on the L‐strand, others were located on the H‐strand (Figure 1). The *ND5* represented the largest PCGs (1839 bp; 612 AA), while the *ATP8* was the smallest (168 bp; 55 AA). All PCGs of *N. argenteus* , with the exception of *COX1*, initiated with methionine (ATG) as the start codon. Notably, *COX1* employed GTG as its start codon. This pattern‐ATG start for most PCGs and GTG for *COX1*—is a conserved feature of vertebrate mitochondrial genomes and has been widely reported in teleost fishes (Tang et al. 2023; Baraf et al. 2024).

Most protein‐coding genes (PCGs) in *N. argenteus* contained complete termination codons TAA (*ND1*, *ND2*, *COX1*, *ATP8*, *ND4L*, and *ND5*) or AGG (*ND6*). Remaining genes had incomplete codons (*COX2*, *ATP6*, *COX3* were TA‐; *ND3*, *ND4*, *Cyt b* were T‐) (Table 2). Significant variability was observed in stop codons of fish mitochondrial genomes, suggesting they may be subjected to a rapid evolutionary process (Peng et al. 2006). Incomplete stop codons were believed to be rectified through a post‐transcriptional polyadenylation mechanism, a process that was widely conserved within the mitochondrial genomes of vertebrate species (Gong et al. 2017).

The base composition of PCGs was biased, with a total AT and GC contents of 56.31% and 43.69%, respectively (Table S2). *ATP8* displayed the highest AT content (60.12%), contrasting with *ND4L*, which exhibited minimal AT content (51.18%). Looking at the base composition of codons, the third position of codons had the highest adenine proportion (34.91%) and guanine was the lowest (10.08%), indicating a clear anti‐G bias. According to the analysis using MEGA software, the mitochondrial protein‐coding genes of *N. argenteus* exhibited a preference for AT (Table S2). Moreover, the *ND6* gene had the highest absolute value of AT‐skew (−0.49), and its GC‐skew was the only positive value (+0.50) among all PCGs. This unique skew pattern of *ND6* has been widely observed across teleost fishes and is attributable to its location on the L‐strand, which subjects it to different mutational pressures during replication compared to H‐strand encoded genes (Tang et al. 2023; Zhang and Cheng 2012). The *ND6* subunit, a functional component of complex I (proton‐translocating NADH‐quinone oxidoreductase), contributes to electron transfer in the NADH respiratory chain and may be subject to distinctive mutational pressures related to its strand location.

Among the PCGs in *N. argenteus* , leucine (17.36%), alanine (8.68%), and threonine (8.16%) were the predominant amino acids, whereas cysteine (Cys) showed the lowest abundance, making up only 0.60% of the total (Table S3). Similar codon usage patterns have been recorded in the other coral reef fishes (Li et al. 2015). Among these amino acids, leucine was used most frequently, possibly because mitochondrial gene‐encoded transmembrane proteins are primarily composed of hydrophobic amino acids.

### 3.4 Non‐Coding Region

The non‐coding regions of the mitochondrial genome primarily regulate gene expression and also play an important role in mitochondrial DNA replication and transcription. The non‐coding regions of the *N. argenteus* mitochondrial gene were similar to that of most fish mitochondrial genes, mainly including the control region (CR) and the L‐strand replication initiation region (O_L_) (Figure 1).

**FIGURE 2:**
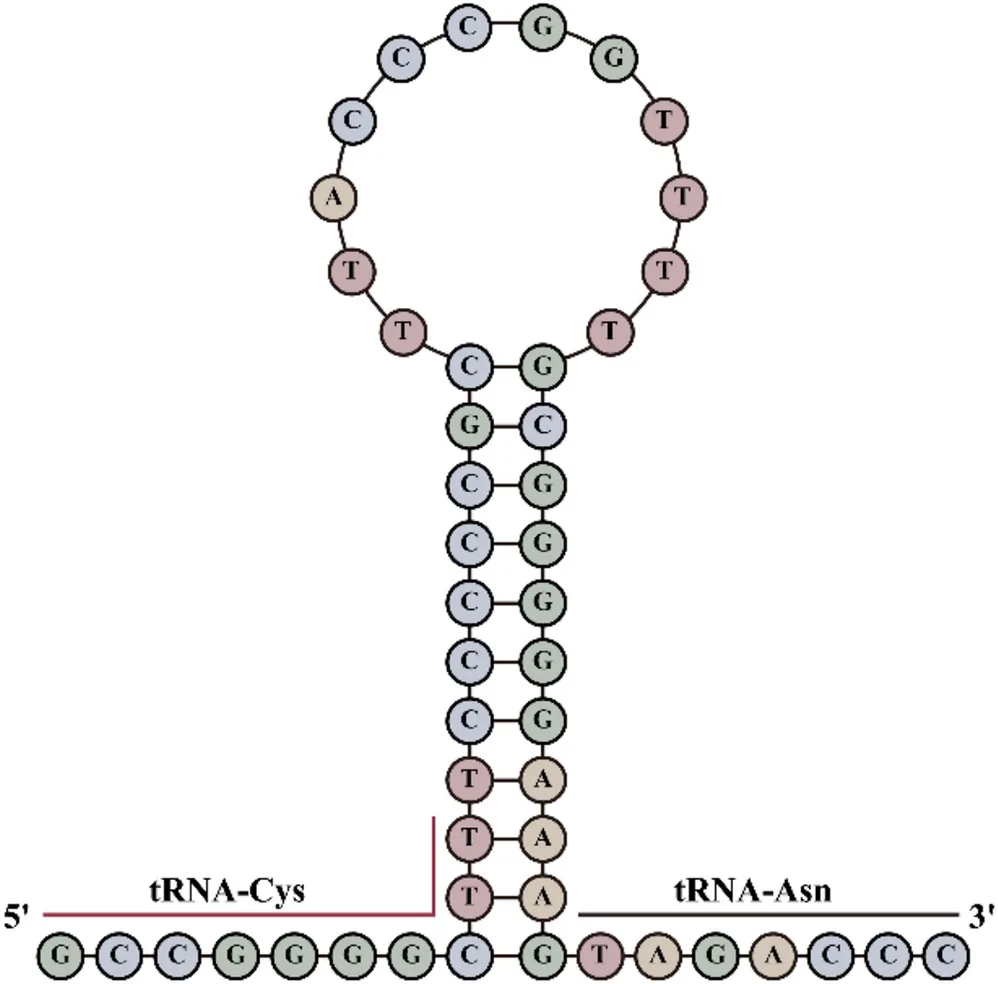
Predicted stem‐loop secondary structure of O_L_ in *Neoniphon argenteus*. The free energy of this O_L_ secondary structure is Δ*G* = −15.3 kcal/mol at 37°C, 1 M Na^+^.

The O_L_ region was positioned between tRNA‐Asn and tRNA‐Cys within the WANCY region (composed of a concatenation of 5 tRNA genes). The O_L_ region, along with parts of the tRNA‐Asn and tRNA‐Cys genes, formed a stable structure consisting of a stem and a loop, with the stem consisting of 11 base pairs and the loop comprising 12 base pairs (Figure 2). At the 5′ end of this stem‐loop structure (tRNA‐Cys gene side), there was a conserved motif (5′‐GCCGG‐3′), which was crucial for L‐strand replication and has also been found in other fish (Brown et al. 2005).

**FIGURE 3:**
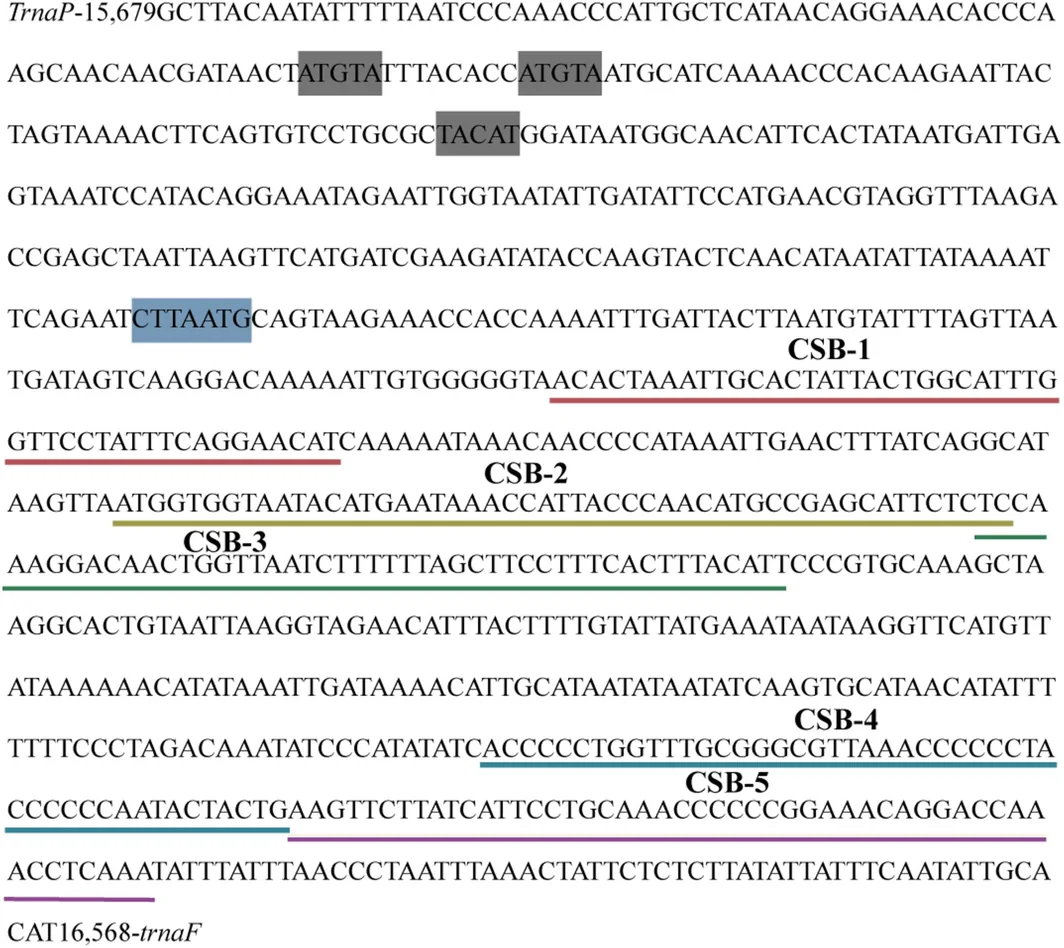
Features present in control regions of the *Neoniphon argenteus* mitogenome. The conserved motifs ATGTA and its complement TACAT are highlighted against a gray background. Additionally, the recurring motifs (CTTAATG) are set against a blue background.

The control region was the fastest evolving sequence in fish mitochondrial DNA, and was widely used in the study of fish population genetics and molecular systematic. The length of control region in *N. argenteus* was 890 bp, with the highest A + T content (66.85%) and exhibited a positive AT‐skew (0.10) and a negative GC‐skew (−0.22) (Table S2), which is consistent with other vertebrates (Sbisa et al. 1997). By aligning of control regions, five conserved sequence blocks (CSB) were detected, namely CSB‐1, CSB‐2, CSB‐3, CSB‐4, and CSB‐5 (Figure 3). Each Conserved Sequence Block (CSB) region exhibited a distinct base composition. Specifically, CSB‐1 was rich in thymine (T) and adenine (A), CSB‐2 was abundant in adenine (A), thymine (T), and cytosine (C), CSB‐3 demonstrated a high concentration of thymine (T), CSB‐4 was cytosine (C)‐rich, and CSB‐5 was characterized by a high presence of adenine (A) and guanine (G) (Table S4). To further examine the conservation of these CSB elements across Holocentridae, we aligned the control region sequences of *N. argenteus* with 14 other holocentrid species. The alignment confirmed that the five CSBs are highly conserved across all examined species, with *N. argenteus* sharing nearly identical CSB sequences with its congener *N. sammara* (Figure S4). Moreover, multiple palindromic sequence motifs (TACAT/ATGTA) with hairpin‐forming capability were identified throughout the control region. These palindromic sequences serve as H‐strand termination sites (Saccone et al. 1999). Similar functional elements recurs in other coral reef fishes (Li et al. 2015).

### 3.5 Codon Usage Patterns

**FIGURE 4:**
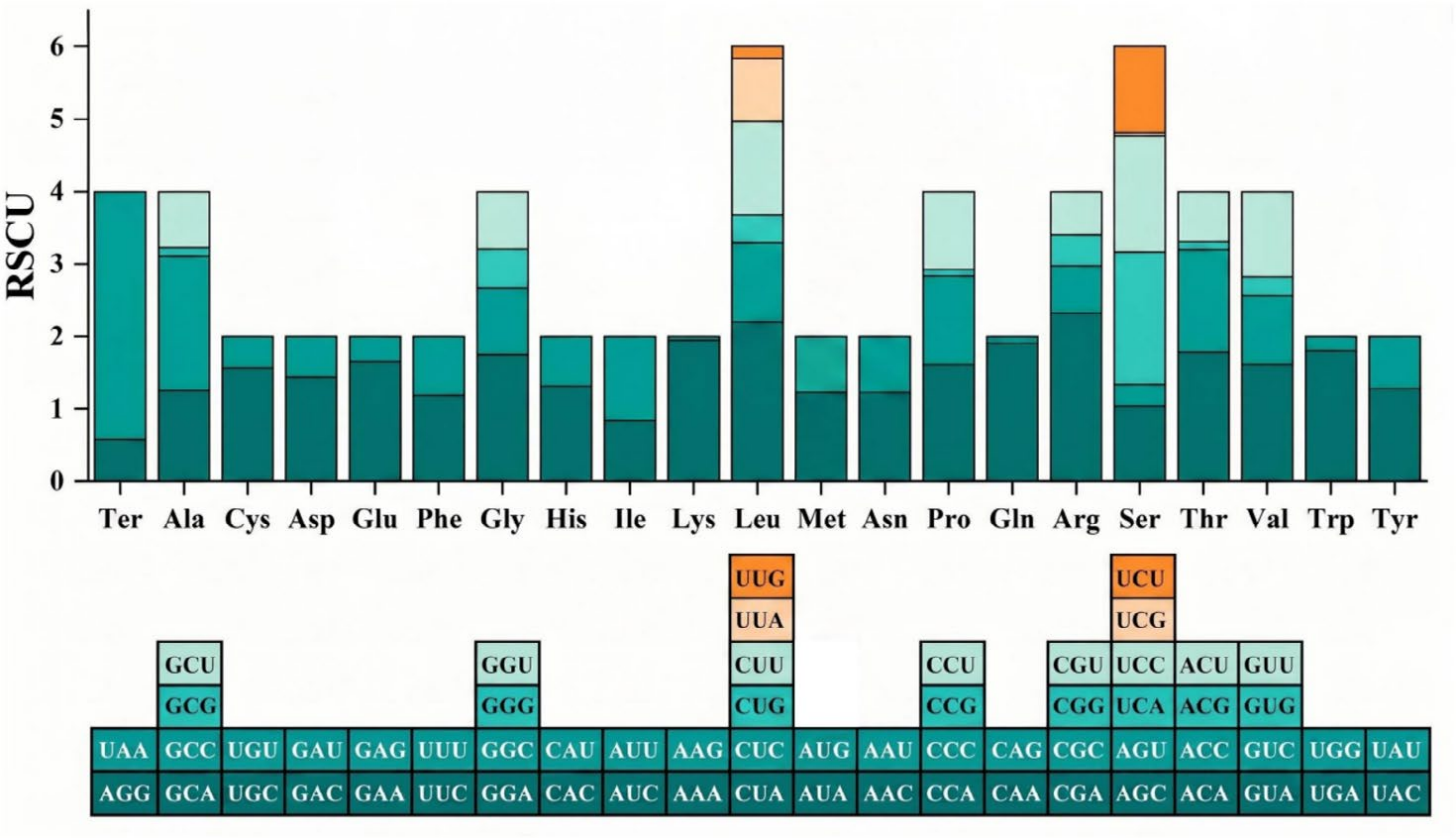
Relative synonymous codon usage (RSCU) of the 13 protein‐coding genes present within the mitochondrial genomes of *Neoniphon argenteus* .

Relative synonymous codon usage (RSCU) serves as a widely used metric for quantifying codon usage bias, with values greater than 1 indicating codons used more frequently than expected under equal synonymous codon usage (Sharp et al. 1986). Analysis of the 13 protein‐coding genes in the mitogenome of *N. argenteus* revealed a total of 31 codons with RSCU > 1 (Figure 4; Table S3). These preferentially used codons showed a marked preference for adenine (A) or cytosine (C) at the third codon position. Examination of the third codon position across synonymous codon families revealed a pronounced preference for adenine, consistent with the overall A + T bias observed throughout the mitogenome (Table S2). Similar patterns of third‐position adenine preference have been documented in other teleost lineages, including members of the family Characidae (Liu et al. 2020) and flatfish (Tan et al. 2025).

The effective number of codons (ENC) values for individual PCGs ranged from 33.72 (*ND6*) to 46.63 (*COX2*), with a mean ENC of 42.15 across all 13 genes (Table 3). ENC values typically range from 20 (extreme bias, where only one codon is used per amino acid) to 61 (no bias, where all synonymous codons are used equally) (Wright 1990). The observed ENC values therefore indicate moderate codon usage bias across the mitogenome. Among the 13 PCGs, *ND6* exhibited the strongest codon bias (ENC = 33.72), followed by *ND4L* (ENC = 37.64), while *COX2* (ENC = 46.63), *ND5* (ENC = 46.33), and *ND3* (ENC = 45.86) displayed relatively weaker bias. A comparison of RSCU values between *COX2* and *ND6* is provided in Table S5.

**TABLE 3:** ENC and GC content at each codon position across 13 PCGs of *Neoniphon argenteus* .

Genes	ENC	GC_all_ (%)	GC_1_ (%)	GC_2_ (%)	GC_3_ (%)
*ND1*	42.35	44.92	57.23	40.62	36.92
*ND2*	42.95	45.65	51.29	44.41	41.26
*COX1*	42.46	41.59	52.42	41.01	31.33
*COX2*	46.63	44.02	55.70	34.60	41.77
*ATP8*	45.41	39.88	46.43	44.64	28.57
*ATP6*	44.85	41.37	56.14	38.60	29.39
*COX3*	38.98	45.42	54.96	42.37	38.93
*ND3*	44.02	44.73	54.70	36.75	42.74
*ND4L*	37.64	48.82	55.56	46.46	44.44
*ND4*	46.17	41.70	49.78	41.99	33.33
*ND5*	42.47	43.99	49.43	39.64	42.90
*ND6*	33.72	44.25	35.06	44.25	53.45
*Cyt b*	40.28	43.73	53.02	39.21	38.85

To further evaluate the codon usage characteristics of *N. argenteus* , we compared its RSCU profile with that of its congener *N. sammara* . The two species showed highly similar RSCU patterns across all codon families, both exhibiting strong preferences for A‐ and C‐ending codons (Figure S3). This similarity is consistent with their close phylogenetic relationship and suggests that codon usage bias is largely conserved within the genus *Neoniphon*.

Analysis of GC content across codon positions revealed a mean GC_1_, GC_2_, and GC_3_ of 48.32%, 39.37%, and 43.36%, respectively (Table 3). The reduced GC content at the second codon position is consistent with the functional constraints imposed on amino acid sequences, as non‐synonymous substitutions at this position are more likely to alter protein structure and function. The GC_3_ values varied substantially among genes, ranging from 28.57% (*ATP8*) to 53.45% (*ND4L*), reflecting gene‐specific mutational pressures and selective constraints. The GC_all_ across all PCGs was 43.68%, further supporting the overall A + T richness characteristic of teleost mitochondrial genomes.

Among the encoded amino acids in *N. argenteus* PCGs, leucine was the most abundant (17.36%), followed by alanine (8.68%) and threonine (8.16%), whereas cysteine showed the lowest frequency (0.60%) (Table S3). The high proportion of leucine, a hydrophobic amino acid, is a common feature of mitochondrial proteomes and likely reflects the predominance of hydrophobic transmembrane domains in mitochondrially encoded proteins (Li et al. 2015). The distribution of codon usage across amino acid families (Figure 4) further illustrates the non‐random utilization of synonymous codons, with certain codons (e.g., CUA for leucine, GCA for alanine, AGA for arginine) being strongly favored over their synonymous alternatives.

To identify putatively optimal codons, we compared RSCU values between high‐bias genes (lower ENC, e.g., *ND6*) and low‐bias genes (higher ENC, e.g., *COX2*). Using the ΔRSCU method (Sharp et al. 1986), we identified 10 candidate optimal codons: GCA (Ala), CAA (Gln), GAA (Glu), GGA (Gly), AUU (Ile), CUA (Leu), CCA (Pro), CGA (Arg), ACA (Thr), and GUC (Val) (Table S5). Notably, nine of the ten candidate optimal codons terminate with adenine (A) or cytosine (C), with AUU (Ile) being the exception (U‐ending), consistent with the overall A + T bias of the *N. argenteus* mitogenome and the preference for A/C‐ending codons observed in other teleost fishes (Zhang et al. 2024; Tan et al. 2025). It should be noted that *ND6* is encoded on the light strand while *COX2* belongs to heavy strand, resulting in distinct strand‐specific mutational bias between the two genes. The ΔRSCU differences observed simultaneously reflect mutational pressure and translational selection rather than purely translation‐associated codon preference. Since this comparison only relies on a single representative gene pair out of 13 mitochondrial PCGs, the 10 identified optimal codons are preliminary candidates and should be interpreted with caution.

Collectively, these codon usage patterns are consistent with those reported for other coral reef fishes (Li et al. 2015) and reflect the combined effects of mutational bias, natural selection, and genetic drift acting on the *N. argenteus* mitochondrial genome. The observed preference for A/C‐ending codons aligns with the high A + T content of the mitogenome, while gene‐specific variations in ENC and GC content suggest that selective constraints differ among the 13 PCGs, likely reflecting their distinct functional roles in oxidative phosphorylation.

### 3.6 Selection Pressure on the Mitochondrial Genome

**FIGURE 5:**
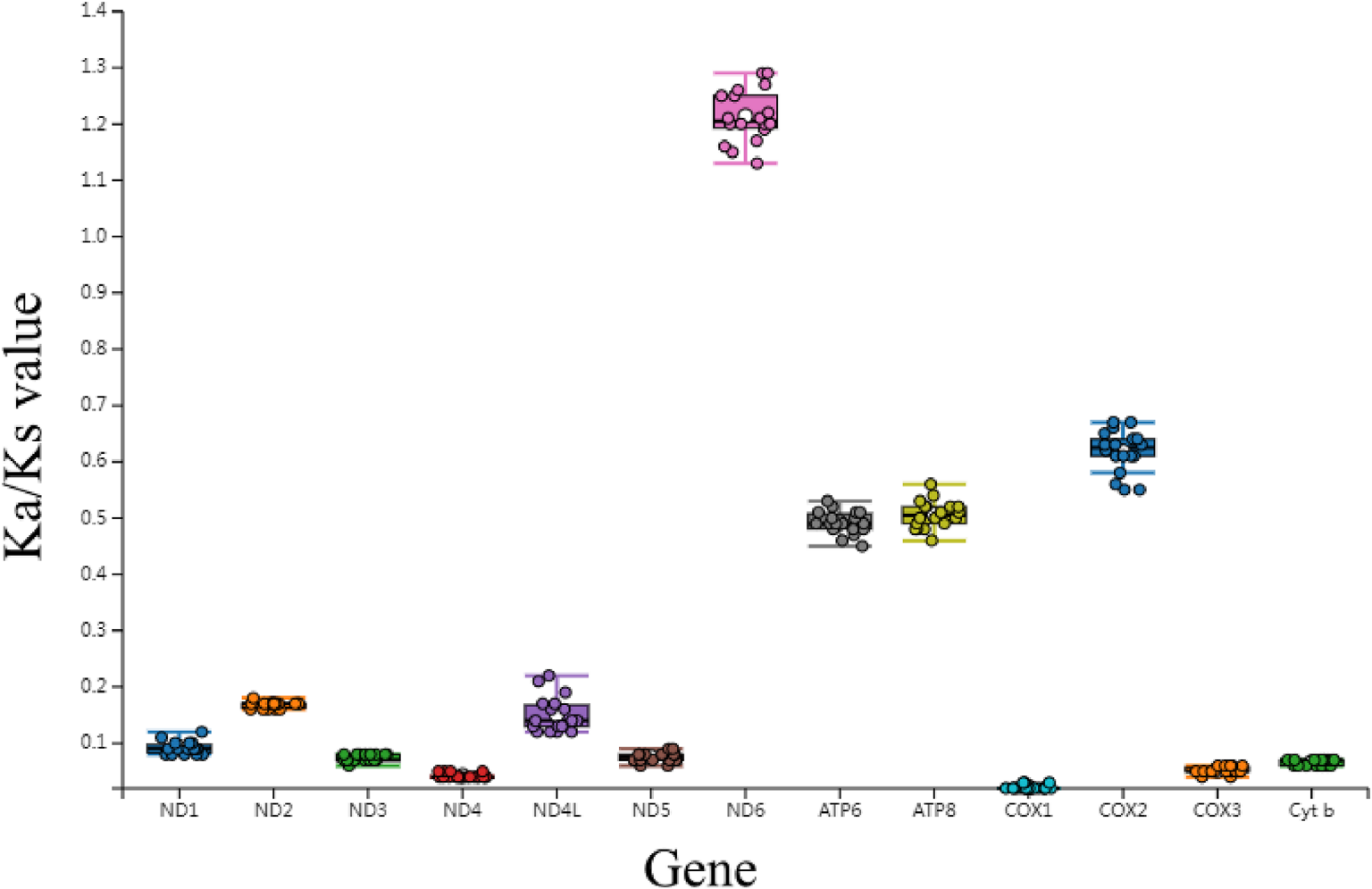
Selection pressure analysis of mitochondrial genes in Holocentriformes species. Error bars represent standard deviations across 18 pairwise comparisons per gene.

The ratio of nonsynonymous to synonymous substitution rates (Ka/Ks, also expressed as *ω* = dN/dS) is a widely used indicator of selective pressure acting on protein‐coding genes, with *ω* < 1 indicating purifying selection, *ω* ≈ 1 suggesting neutral evolution, and *ω* > 1 providing evidence of positive selection (Yang 2007). The Ka/Ks ratio was calculated for 13 PCGs across pairwise comparisons between *N. argenteus* and 18 other species of the order Holocentriformes. Across all 13 PCGs, mean Ka/Ks ratios ranged from 0.022 ± 0.004 (*COX1*) to 1.209 ± 0.047 (*ND6*) (mean ± SD across 18 pairwise comparisons; Figure 5, Table S6). For 12 of the 13 PCGs, Ka/Ks values were consistently less than 1 in all pairwise comparisons, indicating that these genes have been subject to strong purifying selection throughout the evolutionary history of Holocentriformes. The lowest Ka/Ks ratios were observed in *COX1* (mean = 0.022, range = 0.02–0.03), *ND4* (mean = 0.042, range = 0.04–0.05), and *COX3* (mean = 0.052, range = 0.04–0.06), consistent with the high evolutionary conservation of cytochrome c oxidase subunits in teleost fishes (Zhang and Cheng 2012). In contrast, *ND6* exhibited Ka/Ks values greater than 1 in all 18 pairwise comparisons, with a mean of 1.209 ± 0.047 and a range of 1.13–1.29 (Figure 5). *ATP8* also showed an elevated mean Ka/Ks of 0.507 ± 0.027 (range 0.46–0.56), consistent with the commonly observed higher evolutionary rate of this gene (Table S6).

To determine whether the elevated Ka/Ks values observed for *ND6* reflect statistically significant positive selection at specific amino acid sites, site‐specific selection analysis was performed using EasyCodeML (Gao et al. 2019). Likelihood ratio tests comparing nested site models were conducted on the concatenated alignment of all 13 PCGs from 19 Holocentriformes species. No significant differences were detected for either comparison (M1a vs. M2a: 2Δℓ = 0.89, df = 2, *p* > 0.05; M7 vs. M8: 2Δℓ = 0.67, df = 2, *p* > 0.05). Bayes Empirical Bayes (BEB) analysis did not identify any individual codon sites with a posterior probability ≥ 0.95 of being under positive selection. These results indicate that, despite the elevated pairwise Ka/Ks ratios observed for *ND6*, there is no statistical support for pervasive positive selection acting on specific amino acid residues in this gene across the Holocentriformes phylogeny.

Saturation tests using DAMBE confirmed that substitutions in *ND6* had not reached saturation (Iss = 0.412, critical Iss.c = 0.786, *p* < 0.001), and the use of Sanger sequencing effectively ruled out NUMT co‐amplification as a confounding factor.

Taken together, the Ka/Ks analysis demonstrates that all 13 mitochondrial PCGs in *N. argenteus* and related Holocentriformes have been predominantly shaped by purifying selection, consistent with the essential metabolic functions of the proteins they encode (da Fonseca et al. 2008). The elevated Ka/Ks ratio observed for *ND6* in pairwise comparisons likely reflects reduced selective constraint rather than adaptive evolution. This interpretation is supported by the absence of significant positive selection in the site‐specific LRTs and by recent work demonstrating that elevated dN/dS ratios can result from relaxed purifying selection rather than adaptive evolution (Zwonitzer et al. 2022; Baraf et al. 2024). In teleost mitogenomes, *ND6* and *ATP8* frequently exhibit the highest Ka/Ks ratios among PCGs, a pattern consistently interpreted as reflecting relaxation of purifying selection constraints (Baraf et al. 2024; Tang et al. 2023). Furthermore, *ND6* is the only mitochondrial gene encoded on the light strand in vertebrates, and its unique strand‐specific mutational environment has been shown to result in less efficient purifying selection compared to H‐strand encoded genes (Jobson et al. 2010). Similar interpretations have been applied to other teleost lineages, including Engraulidae (Zhang and Cheng 2012) and *Takifugu* (Zhou et al. 2020).

### 3.7 Phylogenetic Tree Analysis

**FIGURE 6:**
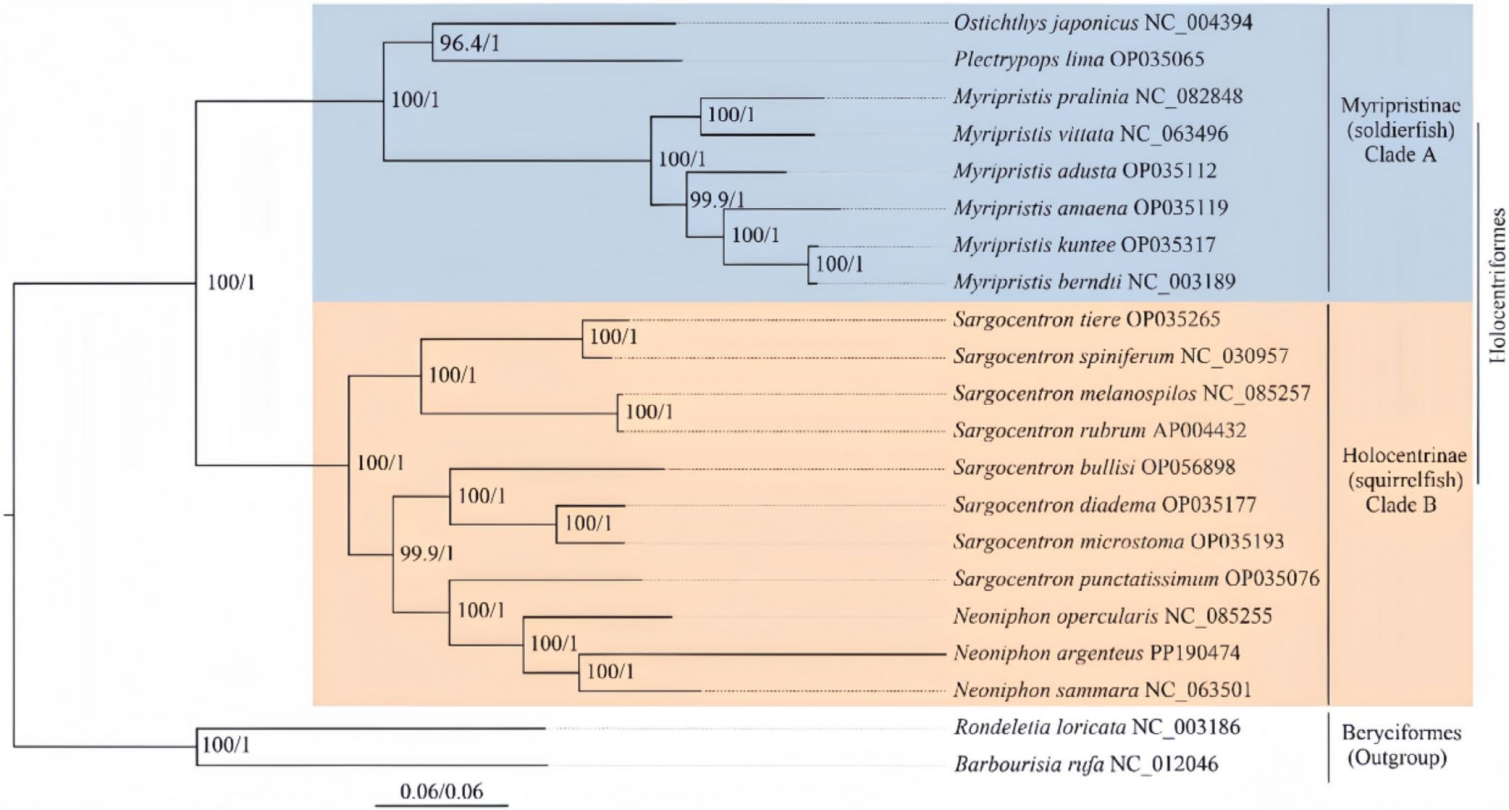
Phylogenetic tree of Holocentriformes species inferred from the concatenated nucleotide sequences of 13 mitochondrial protein‐coding genes (PCGs) using maximum likelihood (ML) and Bayesian inference (BI) methods. *Rondeletia loricata* (NC_003186) and *Barbourisia rufa* (NC_012046) were used as outgroups. Numbers above branches indicate ML bootstrap support values (left; based on 1000 replicates) and Bayesian posterior probabilities (right; based on 10 million MCMC generations). A partitioned analysis strategy was employed, with the concatenated dataset divided into 39 partitions (13 genes × 3 codon positions) and the best‐fit substitution model selected for each partition using ModelTest‐NG. A robustness check including the two rRNA genes (12S and 16S) recovered an identical topology (see Section 3.7).

The partitioned analysis yielded a log‐likelihood of −lnL = 45,673.8, confirming that the partitioned approach better accounts for the heterogeneity in substitution patterns among genes and codon positions. The resulting topologies were highly congruent between the two analytical approaches (Figure 6). Both methods recovered two well‐supported major clades: Clade A and Clade B. Clade A comprised the genera *Ostichthys*, *Plectryops*, and *Myripristis*, corresponding to the subfamily Myripristinae. Clade B contained the genera *Sargocentron* and *Neoniphon*, corresponding to the subfamily Holocentrinae. The monophyly of each subfamily received strong statistical support (BI posterior probability = 1, ML bootstrap = 100), consistent with established morphological and ecological distinctions between squirrelfishes (Holocentrinae) and soldierfishes (Myripristinae) (Greenfield et al. 2017; Fogg et al. 2022).

Maximum likelihood analysis with 1000 bootstrap replicates recovered the same topology as the Bayesian inference, with strong support for all major nodes. The *Ostichthys*–*Plectryops* sister relationship received 96.4% bootstrap support, while all other major nodes received 100% bootstrap support (Figure 6). All major nodes received Bayesian posterior probabilities of 1.00.

As a robustness check, we performed phylogenetic analyses using the expanded dataset of 13 PCGs + 2 rRNAs (15 genes, 13,482 bp). The topology was fully congruent with the 13‐PCGs‐only analysis, with no changes in any major clade or species placement. In the expanded dataset, the *Ostichthys*–*Plectryops* sister relationship received 97.2% bootstrap support (BI = 1.00). All other major nodes received 100% bootstrap support and 1.00 BI posterior probability. The analysis based on deduced amino acid sequences recovered an identical topology, with all major nodes receiving 100% bootstrap support.

Within Clade B (Holocentrinae), *Neoniphon* species formed a monophyletic clade nested within a larger assemblage of *Sargocentron* species with strong support (BI posterior probability = 1, ML bootstrap = 100). This topology indicates that *Sargocentron* as currently defined is paraphyletic with respect to *Neoniphon*, and is consistent with the paraphyletic relationship reported in previous molecular studies (Dornburg et al. 2012; Tang et al. 2023). Specifically, the monophyletic *Neoniphon* clade is nested inside the *Sargocentron* radiation, rendering the genus *Sargocentron* paraphyletic as currently circumscribed. This pattern suggests that the morphological characters traditionally used to distinguish *Neoniphon* from *Sargocentron* may represent derived traits that do not reflect shared common ancestry.

Within Clade A (Myripristinae), *Ostichthys* and *Plectryops* formed a well‐supported clade sister to the genus *Myripristis* (BS = 96.4, BI = 1.00). This topology aligns with earlier systematic hypotheses placing *Plectryops* as closely allied to *Ostichthys* and *Corniger* (Greenfield 1974; Dornburg et al. 2012). Within *Myripristis*, *M. vittata* was deeply nested among congeners, a finding that does not support the monophyly of the subgenus *Archeaomyripristis* as previously proposed on morphological grounds (Greenfield 1974). These discrepancies underscore the complex evolutionary history of Holocentriformes and highlight the utility of mitogenomic data in refining taxonomic hypotheses.

Phylogenetic analysis based on the concatenated amino acid sequences of the 13 PCGs recovered a topology (Figure S2) that was fully congruent with the nucleotide‐based tree (Figure 6). The same two major clades corresponding to Holocentrinae and Myripristinae were recovered with strong support, and the placement of *N. argenteus* within the *Sargocentron* radiation was identical. The congruence between nucleotide‐ and amino acid‐derived phylogenies provides additional confidence in the robustness of the inferred evolutionary relationships among the Holocentriformes species examined.

### 3.8 Limitations of the Study

This study has several limitations. The phylogenetic analysis presented here was based primarily on the mitochondrial genome, a single maternally inherited locus. Gene trees inferred from mitochondrial DNA do not always reflect the underlying species tree, as incomplete lineage sorting of ancestral polymorphisms or historical introgression can lead to discordance between mitochondrial and nuclear phylogenies (Moore 1995; Wiens et al. 2010). The discrepancies observed in this study between the mitogenomic phylogeny and earlier morphology‐based taxonomic hypotheses, such as the paraphyletic relationship between *Neoniphon* and *Sargocentron* and the nested placement of *M. vittata* within *Myripristis*, may therefore stem, at least in part, from the inherent constraints of single‐locus inference. Future work incorporating multiple independent nuclear markers will be necessary to resolve these conflicts with greater confidence. In addition, the selection pressure analysis was conducted on the currently available taxon sampling of 19 Holocentriformes species; broader sampling may reveal lineage‐specific selective pressures not captured in the present dataset. Finally, the absence of *Holocentrus* species from the analysis limits the resolution of relationships within the Holocentrinae clade. Additionally, the 22 tRNA genes were not included in the phylogenetic robustness check due to their short length (60–75 bp), complex secondary structures, and alignment ambiguity, which limited their phylogenetic informativeness. Despite these constraints, the complete mitogenome of *N. argenteus* reported here provides a valuable foundation for future comparative genomic studies of Holocentriformes.

## 4 Conclusions

The current study presents the first complete mitogenome of *N. argenteus* , which comprises 37 distinct mitochondrial genes commonly found in fishes. The composition of the mitogenome in *N. argenteus* exhibited a high degree of similarity to that of other members within the order Holocentriformes. Additionally, we characterized synonymous codon usage patterns across PCGs and detected significant bias, identifying 31 frequently used codons (RSCU > 1) and 10 candidate optimal codons (GCA, CAA, GAA, GGA, AUU, CUA, CCA, CGA, ACA, and GUC). Phylogenetic analysis based on both nucleotide and amino acid datasets, with partitioned models and including rRNA genes as a robustness check, indicated that the Holocentriformes species clustered and formed two strongly supported clades, which belong to the two subfamilies, Holocentrinae and Myripristinae, respectively. Our results confirm that *Neoniphon* forms a monophyletic clade nested within a paraphyletic *Sargocentron*, supporting the paraphyletic relationship reported in previous studies. Selection pressure analysis indicated that all PCGs are predominantly under purifying selection, with no statistically significant evidence of pervasive positive selection. The elevated Ka/Ks ratio observed in *ND6* (mean = 1.209 ± 0.047) likely reflects reduced selective constraint associated with its L‐strand location rather than adaptive evolution, a pattern also observed for *ATP8* (mean = 0.507 ± 0.027). These findings provide useful baseline data for understanding the evolutionary biology and population genetic diversity of *N. argenteus* and offer a foundation for future comparative genomic studies of Holocentriformes.

## Author Contributions

**Lei Xie:** conceptualization (lead), data curation (lead), formal analysis (lead), investigation (lead), writing – original draft (lead). **Nan Chen:** data curation (supporting), investigation (supporting), methodology (supporting). **Qi Qiao:** data curation (supporting), methodology (supporting), resources (lead). **Xuedong Xie:** data curation (supporting), formal analysis (supporting). **Bozhu Huang:** investigation (supporting), validation (supporting). **Songhui Lu:** funding acquisition (supporting), supervision (supporting). **Yuelei Dong:** methodology (lead), supervision (supporting), validation (supporting). **Lei Cui:** funding acquisition (lead), supervision (lead), validation (supporting).

## Funding

This work was supported by the National Natural Science Foundation of China (No. 42176142) and Construction of a Typical Water Source Floating Plant Biodiversity Database Based on Environmental DNA Technology (GDEEMC‐2024‐12).

## Conflicts of Interest

The authors declare no conflicts of interest.

## Supporting information


**Table S1:** Primer designed according to the mitochondrial genome sequence of *N. sammara* .
**Table S2:** Base composition of mitochondrial protein‐coding genes (PCGs), tRNA, rRNA, and D‐loop of *N. argenteus* .
**Table S3:** Codon usage frequency of mitochondrial encoded protein in *N. argenteus* .
**Table S4:** The base composition of the CSBs of the control region of *N. argenteus* mitogenome.
**Table S5:** ΔRSCU values between high‐expression gene (*COX2*) and low‐expression gene (*ND6*) for candidate optimal codon identification.
**Table S6:** Summary of Ka/Ks ratios for 13 mitochondrial protein‐coding genes calculated from pairwise comparisons between *Neoniphon argenteus* and 18 other Holocentriformes species.
**Figure S1:** Second structures of transfer RNAs in the *N. argenteus* mitogenome.
**Figure S2:** Phylogenetic tree of Holocentriformes species inferred from the concatenated amino acid sequences of 13 mitochondrial genes.
**Figure S3:** Comparison of relative synonymous codon usage (RSCU) between *N. argenteus* and *N. sammara* based on 13 mitochondrial protein‐coding genes. RSCU values were calculated using the vertebrate mitochondrial genetic code (NCBI translation Table 2). The dashed gray line at *y* = 1 indicates equal synonymous codon usage (no bias). Codons with RSCU > 1 are used more frequently than expected.
**Figure S4:** Alignment of conserved sequence blocks (CSB‐I to CSB‐V) in the control regions of Holocentridae species.

## Data Availability

All the required data are uploaded as Supporting Information. The complete mitochondrial genome of *Neoniphon argenteus* was deposited in the GenBank of NCBI (https://www.ncbi.nlm.nih.gov) and obtained the accession number (GenBank accession no. PP190474.1). The original Sanger sequencing chromatograms and the sequence alignments used for phylogenetic and selection analyses have been uploaded into Dryad under the following https://doi.org/10.5061/dryad.vhhmqqp8h. The voucher specimen (ID: NH2023‐04) is deposited in the laboratory of the Department of Biology, Jinan University.
