# Supplementary material for: Insights Into the Structural Features, Codon Usage Patterns, and Phylogenetic Analysis in Neoniphon argenteus (Teleostei: Holocentriformes) Based on Complete Mitochondrial Genome
Source: Ecol Evol. 2026 Sep 4;16(9):e74291. doi: 10.1002/ece3.74291 (PMC13543163; doi:10.1002/ece3.74291)
Supplement: Supplementary file 1 [file ECE3-16-e74291-s001.doc]

Insights into the structural features, codon usage patterns and phylogenetic analysis in *Neoniphon argenteus* (Teleostei: Holocentriformes) based on complete mitochondrial genome

Lei Xie ^1)^, Nan Chen ^1)^, Qi Qiao ^1)^, Xuedong Xie ^3)^, Bozhu Huang ^3)^, Songhui Lu ^1), *^,

Yuelei Dong ^1), 2), *^, Lei Cui ^1), *^

*^1^ Key Laboratory of Eutrophication and Red Tide Prevention of Guangdong Higher Education Institutes, College of Life Science and Technology, and Southern Marine Science and Engineering Guangdong Laboratory (Zhuhai), Jinan University, Guangzhou 510632, China.*

*^2^ Guangdong Provincial Key Laboratory of Biotechnology for Plant Development, School of Life Sciences, South China Normal University, Guangzhou 510631, China.*

*^3^ Guangdong Ecological and Environmental Monitoring Center, Guangzhou 510308, China.*

^*^ Corresponding authors:

Dr. Lei Cui

E-mail: leicui@jnu.edu.cn (L.C.)

Dr. Yuelei Dong

E-mail: yueleidong@scnu.edu.cn (Y. D.)

Dr. Songhui Lu

E-mail: lusonghui1963@163.com (S. L.)

***Table S1. Primer designed according to the mitochondrial genome sequence of N. sammara***

| Primer name | Primer sequence | Primer location | Length | Annealing temperature |
| --- | --- | --- | --- | --- |
| 1F | AACTGTTATACGTACCCGAAGGC | 406-428 | 513 | 65.2 |
| 1R | GAGAGTGACGGGCGGTGTGT | 899-918 |  |  |
| 2F | AAACTGGGATTAGATACCCCACTAT | 487-511 | 2151 | 64.1 |
| 2R | CCGGTCTGAACTCAGATCACGT | 2616-2637 |  |  |
| 3F | TTACGACCTCGATGTTGGATCAG | 2536-2558 | 1441 | 60.1 |
| 3R | GGAAGCACTAAGAGTTTTGATC | 3955-3976 |  |  |
| 4F | AAGGGCCACTTTGATAGAGTG | 3871-3891 | 999 | 57.0 |
| 4R | GTAAGTTCTTGTAGGATTAATCA | 4847-4869 |  |  |
| 5F | TAGCATATTCATCAATCGCCCAC | 4593-4615 | 1200 | 57.8 |
| 5R | CTTATGTTATTTATTCGTGGGAA | 5770-5792 |  |  |
| 6F | CCTTTATTTAGTATTCGGTGC | 5541-5561 | 1552 | 56.7 |
| 6R | ATGTGACTGGCTTGAAA | 7076-7092 |  |  |
| 7F | AATACCTGAACTAAAATCCACTT | 6709-6731 | 1439 | 59.0 |
| 7R | AATTGATCGAAGAAGCTTAGTGTCA | 8123-8147 |  |  |
| 8F | AGCGTTAGCCTTTTAAGC | 7912-7929 | 940 | 58.5 |
| 8R | CATGGGCTGGGGTCGACTAT | 8832-8851 |  |  |
| 9F | CCCACCCTTCTAATCCCAGTTCTTAT | 8524-8549 | 1296 | 65.1 |
| 9R | AGAAAAAGGAAGTCGGGCTGT | 9799-9819 |  |  |
| 10F | AGAAAAAGGAAGTCGGGCTGT | 9609-9628 | 2336 | 62.8 |
| 10R | CAAGAGTTTTTGGTTCCTAAGACC | 11921-11944 |  |  |
| 11F | AACAAAAACATTAGATTGTGATTC | 11768-11791 | 2574 | 57.8 |
| 11R | TTAGAGTCCGGGCAGGAATTATG | 14319-14341 |  |  |
| 12F | GGACCAATGACTTGAAAAACCACCGTTG | 14245-14372 | 1750 | 56.7 |
| 12R | TTCAGGAATCAAATTTTGGT | 15975-15944 |  |  |
| 13F | TGGTTCCTATTTCAGGTACAT | 16068-16088 | 1189 | 59.6 |
| 13R | GCATAGTGGGGTATCTAATCCCAGTTTG | 486-513 |  |  |

**Table S2. Base composition of mitochondrial protein-coding genes (PCGs), tRNA, rRNA, and D-loop of *N. argenteus***

| Gene name | Length(bp) | Base composition | | | | | | AT-skew | GC-skew |
| --- | --- | --- | --- | --- | --- | --- | --- | --- | --- |
|  |  | A (%) | T (%) | C (%) | G (%) | (A+T)(%) | (C+G)(%) |  |  |
| *ND1* | 975 | 27.38 | 27.69 | 30.87 | 14.05 | 55.07 | 44.93 | -0.01 | -0.37 |
| *ND2* | 1047 | 30.37 | 23.97 | 35.05 | 10.60 | 54.34 | 45.66 | 0.12 | -0.54 |
| *COX1* | 1551 | 27.60 | 30.82 | 24.63 | 16.96 | 58.42 | 41.58 | -0.06 | -0.19 |
| *COX2* | 692 | 29.91 | 26.16 | 28.32 | 15.61 | 56.07 | 43.93 | 0.07 | -0.29 |
| *ATP8* | 168 | 30.95 | 29.17 | 29.17 | 10.71 | 60.12 | 39.88 | 0.03 | -0.46 |
| *ATP6* | 683 | 27.82 | 30.75 | 30.16 | 11.27 | 58.57 | 41.43 | -0.05 | -0.46 |
| *COX3* | 785 | 27.13 | 26.62 | 29.94 | 16.31 | 53.75 | 46.25 | 0.01 | -0.30 |
| *ND3* | 349 | 24.64 | 30.66 | 30.95 | 13.75 | 55.30 | 44.70 | -0.11 | -0.39 |
| *ND4L* | 297 | 20.20 | 30.98 | 29.63 | 19.19 | 51.18 | 48.82 | -0.21 | -0.21 |
| *ND4* | 1381 | 29.98 | 28.31 | 28.67 | 13.03 | 58.29 | 41.71 | 0.03 | -0.38 |
| *ND5* | 1839 | 30.67 | 25.34 | 31.32 | 12.67 | 56.01 | 43.99 | 0.10 | -0.42 |
| *ND6* | 522 | 14.17 | 41.58 | 11.11 | 33.14 | 55.75 | 44.25 | -0.49 | 0.50 |
| *Cyt b* | 1141 | 27.43 | 28.83 | 29.45 | 14.29 | 56.27 | 43.73 | -0.03 | -0.35 |
| PCGs | 11430 | 29.13 | 27.18 | 29.86 | 13.83 | 56.31 | 43.69 | 0.03 | -0.37 |
| tRNAs | 1556 | 31.17 | 24.87 | 24.61 | 19.34 | 56.04 | 43.96 | 0.11 | -0.12 |
| rRNAs | 2652 | 34.09 | 21.12 | 24.55 | 20.25 | 55.21 | 44.79 | 0.24 | -0.10 |
| Control region | 890 | 36.63 | 30.22 | 20.22 | 12.92 | 66.85 | 33.15 | 0.10 | -0.22 |
| mtDNA | 16569 | 30.48 | 26.13 | 28.02 | 15.37 | 56.61 | 43.39 | 0.08 | -0.29 |

***Table S3. Codon usage frequency of mitochondrial encoded protein in N. argenteus***

| Amino Acid | Codon | Count | RSCU | Amino Acid | Codon | Count | RSCU |
| --- | --- | --- | --- | --- | --- | --- | --- |
| TER | AGA | 0 | 0 | M | AUG | 42 | 0.50 |
| TER | AGG | 1 | 0.57 | N | AAC | 75 | 1.22 |
| TER | UAA | 6 | 3.43 | N | AAU | 48 | 0.78 |
| TER | UAG | 0 | 0 | P | CCA | 88 | 1.61 |
| A | GCA | 103 | 1.25 | P | CCC | 67 | 1.22 |
| A | GCC | 153 | 1.85 | P | CCG | 5 | 0.09 |
| A | GCG | 10 | 0.12 | P | CCU | 59 | 1.08 |
| A | GCU | 64 | 0.78 | Q | CAA | 100 | 1.90 |
| C | UGC | 18 | 1.57 | Q | CAG | 5 | 0.10 |
| C | UGU | 5 | 0.43 | R | CGA | 43 | 2.32 |
| D | GAC | 53 | 1.43 | R | CGC | 12 | 0.65 |
| D | GAU | 21 | 0.57 | R | CGG | 8 | 0.43 |
| E | GAA | 84 | 1.65 | R | CGU | 11 | 0.59 |
| E | GAG | 18 | 0.35 | S | AGC | 40 | 1.03 |
| F | UUC | 136 | 1.18 | S | AGU | 12 | 0.31 |
| F | UUU | 94 | 0.82 | S | UCA | 71 | 1.83 |
| G | GGA | 106 | 1.75 | S | UCC | 62 | 1.60 |
| G | GGC | 55 | 0.91 | S | UCG | 2 | 0.05 |
| G | GGG | 33 | 0.55 | S | UCU | 46 | 1.18 |
| G | GGU | 48 | 0.79 | T | ACA | 138 | 1.78 |
| H | CAC | 69 | 1.31 | T | ACC | 110 | 1.42 |
| H | CAU | 36 | 0.69 | T | ACG | 8 | 0.10 |
| I | AUC | 115 | 0.83 | T | ACU | 54 | 0.70 |
| I | AUU | 162 | 1.17 | V | GUA | 86 | 1.61 |
| K | AAA | 74 | 1.95 | V | GUC | 51 | 0.95 |
| K | AAG | 2 | 0.05 | V | GUG | 14 | 0.26 |
| L | CUA | 242 | 2.20 | V | GUU | 63 | 1.18 |
| L | CUC | 120 | 1.09 | W | UGA | 108 | 1.80 |
| L | CUG | 42 | 0.38 | W | UGG | 12 | 0.20 |
| L | CUU | 142 | 1.29 | Y | UAC | 73 | 1.28 |
| L | UUA | 96 | 0.87 | Y | UAU | 41 | 0.72 |
| L | UUG | 18 | 0.16 |  |  |  |  |
| M | AUA | 126 | 1.50 |  |  |  |  |

**Table S4** The base composition of the CSBs of the control region of *N. argenteus* mitogenome

| Base Composition (%) | CSB-1 | CSB-2 | CSB-3 | CSB-4 | CSB-5 |
| --- | --- | --- | --- | --- | --- |
| A | 29.17 | 34.69 | 24.49 | 17.02 | 36.00 |
| T | 35.42 | 24.49 | 42.86 | 21.28 | 18.00 |
| C | 20.83 | 24.49 | 22.45 | 44.68 | 12.00 |
| G | 14.58 | 16.33 | 10.20 | 17.02 | 54.00 |

**Table S5** ΔRSCU values between high-expression gene (*COX2*) and low-expression gene (*ND6*) for candidate optimal codon identification.

| Amino acid | Codon | High-expression gene (*COX2*) | | Low-expression gene (*ND6*) | | ΔRSCU | Candidate optimal |
| --- | --- | --- | --- | --- | --- | --- | --- |
|  |  | Number | RSCU | Number | RSCU |  |  |
| Ter | AGA | 0 | 0.00 | 9 | 1.44 | -1.44 | No |
| Ter | AGG | 1 | 4.00 | 4 | 0.64 | 3.36 | No |
| Ter | UAA | 0 | 0.00 | 11 | 1.76 | -1.76 | No |
| Ter | UAG | 0 | 0.00 | 1 | 0.16 | -0.16 | No |
| Ala | GCA | 8 | 1.88 | 0 | 0.00 | 1.88 | Yes |
| Ala | GCC | 4 | 0.94 | 1 | 2.00 | -1.06 | No |
| Ala | GCG | 1 | 0.24 | 0 | 0.00 | 0.24 | No |
| Ala | GCU | 4 | 0.94 | 1 | 2.00 | -1.06 | No |
| Cys | UGC | 2 | 2.00 | 2 | 2.00 | 0.00 | No |
| Cys | UGU | 0 | 0.00 | 0 | 0.00 | 0.00 | No |
| Asp | GAC | 7 | 1.08 | 1 | 2.00 | -0.92 | No |
| Asp | GAU | 6 | 0.92 | 0 | 0.00 | 0.92 | No |
| Glu | GAA | 13 | 1.73 | 0 | 0.00 | 1.73 | Yes |
| Glu | GAG | 2 | 0.27 | 0 | 0.00 | 0.27 | No |
| Phe | UUC | 7 | 1.56 | 1 | 2.00 | -0.44 | No |
| Phe | UUU | 2 | 0.44 | 0 | 0.00 | 0.44 | No |
| Gly | GGA | 2 | 0.89 | 0 | 0.00 | 0.89 | Yes |
| Gly | GGC | 4 | 1.78 | 0 | 0.00 | 1.78 | No |
| Gly | GGG | 0 | 0.00 | 0 | 0.00 | 0.00 | No |
| Gly | GGU | 3 | 1.33 | 1 | 4.00 | -2.67 | No |
| His | CAC | 8 | 1.60 | 7 | 1.17 | 0.43 | No |
| His | CAU | 2 | 0.40 | 5 | 0.83 | -0.43 | No |
| Ile | AUC | 8 | 0.73 | 2 | 1.33 | -0.61 | No |
| Ile | AUU | 14 | 1.27 | 1 | 0.67 | 0.60 | Yes |
| Lys | AAA | 4 | 1.60 | 12 | 1.50 | 0.10 | No |
| Lys | AAG | 1 | 0.40 | 4 | 0.50 | -0.10 | No |
| Leu | CUA | 8 | 1.78 | 0 | 0.00 | 1.78 | Yes |
| Leu | CUC | 6 | 1.33 | 5 | 5.00 | -3.67 | No |
| Leu | CUG | 1 | 0.22 | 0 | 0.00 | 0.22 | No |
| Leu | CUU | 7 | 1.56 | 1 | 1.00 | 0.56 | No |
| Leu | UUA | 5 | 1.11 | 0 | 0.00 | 1.11 | No |
| Leu | UUG | 0 | 0.00 | 0 | 0.00 | 0.00 | No |
| Met | AUA | 9 | 1.50 | 10 | 2.00 | -0.50 | No |
| Met | AUG | 3 | 0.50 | 0 | 0.00 | 0.50 | No |
| Asn | AAC | 4 | 1.33 | 13 | 1.63 | -0.29 | No |
| Asn | AAU | 2 | 0.67 | 3 | 0.38 | 0.29 | No |
| Pro | CCA | 8 | 2.29 | 1 | 0.25 | 2.04 | Yes |
| Pro | CCC | 4 | 1.14 | 12 | 3.00 | -1.86 | No |
| Pro | CCG | 0 | 0.00 | 2 | 0.50 | -0.50 | No |
| Pro | CCU | 2 | 0.57 | 1 | 0.25 | 0.32 | No |
| Gln | CAA | 8 | 2.00 | 11 | 1.69 | 0.31 | Yes |
| Gln | CAG | 0 | 0.00 | 2 | 0.31 | -0.31 | No |
| Arg | CGA | 3 | 2.00 | 0 | 0.00 | 2.00 | Yes |
| Arg | CGC | 2 | 1.33 | 6 | 2.67 | -1.33 | No |
| Arg | CGG | 0 | 0.00 | 2 | 0.89 | -0.89 | No |
| Arg | CGU | 1 | 0.67 | 1 | 0.44 | 0.22 | No |
| Ser | AGC | 2 | 0.75 | 7 | 2.10 | -1.35 | No |
| Ser | AGU | 1 | 0.38 | 2 | 0.60 | -0.23 | No |
| Ser | UCA | 5 | 1.88 | 4 | 1.20 | 0.68 | No |
| Ser | UCC | 7 | 2.63 | 4 | 1.20 | 1.43 | No |
| Ser | UCG | 0 | 0.00 | 3 | 0.90 | -0.90 | No |
| Ser | UCU | 1 | 0.38 | 0 | 0.00 | 0.38 | No |
| Thr | ACA | 4 | 1.33 | 1 | 0.31 | 1.03 | Yes |
| Thr | ACC | 4 | 1.33 | 8 | 2.46 | -1.13 | No |
| Thr | ACG | 1 | 0.33 | 0 | 0.00 | 0.33 | No |
| Thr | ACU | 3 | 1.00 | 4 | 1.23 | -0.23 | No |
| Val | GUA | 5 | 1.11 | 1 | 4.00 | -2.89 | No |
| Val | GUC | 7 | 1.56 | 0 | 0.00 | 1.56 | Yes |
| Val | GUG | 4 | 0.89 | 0 | 0.00 | 0.89 | No |
| Val | GUU | 2 | 0.44 | 0 | 0.00 | 0.44 | No |
| Trp | UGA | 5 | 2.00 | 1 | 2.00 | 0.00 | No |
| Trp | UGG | 0 | 0.00 | 0 | 0.00 | 0.00 | No |
| Tyr | UAC | 9 | 1.80 | 6 | 2.00 | -0.20 | No |
| Tyr | UAU | 1 | 0.20 | 0 | 0.00 | 0.20 | No |

*Note: These comparisons are based on a limited dataset of 13 mitochondrial PCGs and should be interpreted with caution.*

**Table S6** Summary of Ka/Ks ratios for 13 mitochondrial protein-coding genes calculated from pairwise comparisons between *Neoniphon argenteus* and 18 other Holocentriformes species.

| Gene | Mean | SD | SE | 95% CI | Range |
| --- | --- | --- | --- | --- | --- |
| *ND1* | 0.091 | 0.011 | 0.0026 | [0.086, 0.096] | 0.08-0.12 |
| *ND2* | 0.168 | 0.005 | 0.0012 | [0.165, 0.171] | 0.16-0.18 |
| *ND3* | 0.072 | 0.007 | 0.0016 | [0.069, 0.075] | 0.06-0.09 |
| *ND4* | 0.041 | 0.004 | 0.0009 | [0.039, 0.043] | 0.04-0.05 |
| *ND4L* | 0.150 | 0.029 | 0.0068 | [0.136, 0.164] | 0.12-0.22 |
| *ND5* | 0.075 | 0.008 | 0.0019 | [0.071, 0.079] | 0.06-0.09 |
| *ND6* | 1.209 | 0.047 | 0.0111 | [1.186, 1.232] | 1.13-1.29 |
| *ATP6* | 0.490 | 0.022 | 0.0052 | [0.479, 0.501] | 0.45-0.53 |
| *ATP8* | 0.507 | 0.027 | 0.0064 | [0.493, 0.521] | 0.46–0.56 |
| *COX1* | 0.021 | 0.003 | 0.0007 | [0.020, 0.022] | 0.02-0.03 |
| *COX2* | 0.611 | 0.036 | 0.0085 | [0.593, 0.629] | 0.55-0.67 |
| *COX3* | 0.050 | 0.006 | 0.0014 | [0.047, 0.053] | 0.04-0.06 |
| *Cyt b* | 0.064 | 0.006 | 0.0014 | [0.061, 0.067] | 0.05-0.07 |

**Fig. S1** Second structures of transfer RNAs in the *N. argenteus* mitogenome.

**Fig. S2** Phylogenetic tree of Holocentriformes species inferred from the concatenated amino acid sequences of 13 mitochondrial genes.

**Fig. S3** Comparison of relative synonymous codon usage (RSCU) between *N. argenteus* and *N. sammara* based on 13 mitochondrial protein-coding genes. RSCU values were calculated using the vertebrate mitochondrial genetic code (NCBI translation table 2). The dashed gray line at y = 1 indicates equal synonymous codon usage (no bias). Codons with RSCU > 1 are used more frequently than expected.

**Fig. S4** Alignment of conserved sequence blocks (CSB-I to CSB-V) in the control regions of Holocentridae species.
